# Developing Digital Therapeutics for Chronic Pain in Primary Care: A Qualitative Human-Centered Design Study of Providers’ Motivations and Challenges

**DOI:** 10.2196/41788

**Published:** 2023-02-03

**Authors:** Kris Pui Kwan Ma, Kari A Stephens, Rachel E Geyer, Maria G Prado, Brenda L Mollis, Susan M Zbikowski, Deanna Waters, Jo Masterson, Ying Zhang

**Affiliations:** 1 Department of Family Medicine University of Washington Seattle, WA United States; 2 2Morrow Inc Kirkland, WA United States

**Keywords:** chronic pain, pain management, primary care, digital therapeutics, mHealth, mobile health, human-centered design, digital health, pain, qualitative, interview, challenge, perspective, health care provider, physician, doctor

## Abstract

**Background:**

Digital therapeutics are growing as a solution to manage pain for patients; yet, they are underused in primary care where over half of the patients with chronic pain seek care. Little is known about how to successfully engage primary care providers in recommending digital therapeutics to their patients. Exploring provider motivations in chronic pain management would potentially help to improve their engagement and inform the development of digital therapeutics.

**Objective:**

This study examined primary care providers’ motivations for chronic pain management, including their strategies and challenges, to inform the future development of chronic pain-related digital therapeutics tailored to primary care settings.

**Methods:**

We conducted qualitative semistructured interviews with health care providers recruited from 3 primary care clinics in Washington and 1 clinic in Colorado between July and October 2021. The sample (N=11) included 7 primary care physicians, 2 behavioral health providers, 1 physician assistant, and 1 nurse. Most providers worked in clinics affiliated with urban academic health systems. Guided by the human-centered design approach and Christensen’s Job-to-be-Done framework, we asked providers their goals and priorities in chronic pain management, their experiences with challenges and strategies used to care for patients, and their perceptions of applying digital therapeutics in clinical practice. Transcripts were analyzed using a thematic analysis approach.

**Results:**

We found that primary care providers were motivated but challenged to strengthen the patient-provider alliance, provide team-based care, track and monitor patients’ progress, and address social determinants of health in chronic pain management. Specifically, providers desired additional resources to improve patient-centered communication, pain education and counseling, and goal setting with patients. Providers also requested greater accessibility to multidisciplinary care team consultations and nonpharmacological pain treatments. When managing chronic pain at the population level, providers need infrastructure and systems to systematically track and monitor patients’ pain and provide wraparound health and social services for underserved patients. Recommendations on digital therapeutic features that might address provider challenges in achieving these motivations were discussed.

**Conclusions:**

Given the findings, to engage primary care providers, digital therapeutics for chronic pain management need to strengthen the patient-provider alliance, increase access to nonpharmacological treatment options, support population health tracking and management, and provide equitable reach. Leveraging digital therapeutics in a feasible, appropriate, and acceptable way to aid primary care providers in chronic pain management may require multimodal features that address provider motivations at an individual care and clinic or system level.

## Introduction

Chronic pain is defined as persistent or recurrent pain for 3 or more months [1]. About 20% (50 million) of the US adult population experience chronic pain, with 40% (19.6 million) of those people reporting impairments in daily functioning and work life [2]. More than half of patients with chronic pain receive treatment in primary care [3]. Managing chronic pain is complex and challenging for many primary care providers due to short visit times, lack of training, frustrations with prescribing chronic opioids, and high comorbidity with other chronic conditions [4,5]. One in five visits in primary care is for chronic pain and has resulted in overreliance in primary care for prescription opioids, despite evidence-based guidelines recommending non–opiate-based care and therapies [6].

As pharmacologic treatments of pain have limited efficacy and potential side effects and harms, most chronic pain guidelines include nonpharmacologic and complementary therapies to treat and manage chronic pain [7]. Evidence-based approaches include exercise, acupuncture, spinal manipulative therapy, and behavioral interventions [8]. Behavioral interventions are recommended as a core part of primary care treatment of chronic pain, with improvements seen in patient function, pain, mood, depression, and health-related quality of life [9]. Both cognitive behavioral therapy and acceptance and commitment therapy have shown similar efficacy in pain improvements via supporting patients to cope with their maladaptive thoughts, behaviors, and feelings associated with chronic pain [10-12]. Nevertheless, primary care patients experiencing chronic pain have difficulty accessing these nonpharmacologic recommended therapies due to barriers like long wait times, cost, shortage of behavioral health workers, physicians’ inadequate knowledge of therapies, and patients’ limited understanding and skepticism of the effectiveness of therapies [13,14].

Digital therapeutics, evidence-based high-quality software products that deliver therapeutic interventions to treat, manage, and prevent a broad spectrum of diseases [15], are growing as a solution to improve access and quality of care [16,17]. An emerging body of evidence has shown the efficacy of digital therapeutics in decreasing pain intensity and improving quality of life [11,18-20]. However, most app-based digital therapeutics for chronic pain only focus on patient self-management and lack opportunities to communicate with clinicians or peers [21,22]. These chronic pain apps are underused in health care settings, including primary care providers who see over half of the patients with chronic pain [23,24]. Review papers assessing pain management apps have called for more rigorous research with quality testing and inclusive input of end users including health care providers in the app development process [20-22,25]. However, little is known about successfully engaging primary care providers in recommending digital therapeutics to their patients with chronic pain. Therefore, using a qualitative, human-centered design study, we examined primary care providers’ motivations for chronic pain management, including their strategies and challenges, to inform the future development of chronic pain-related digital therapeutics tailored to primary care settings.

## Methods

### Study Design

A human-centered design approach, which grounds the development of any innovations, technologies, and products based on the needs of the people and the settings in which they will use it, was used to develop the study [26]. The goal is to increase the accessibility and usability of the products to target users and maximize user satisfaction. The global design firm IDEO popularized human-centered design and broke down the iterative process into three phases: (1) inspiration—learn from users or customers about what they want; (2) ideation—brainstorm, test, and refine ideas based on users’ feedback; and (3) implementation—bring the ideal solution to the market and maximize its impact [27]. This study is situated in the inspiration phase where we created a process to emphasize with providers, one of the primary end users of chronic pain-related digital therapeutics, and capture details of what they do, feel, and think in day-to-day experiences as well as the contexts in shaping their experiences [26,27]. This process included envisioning multiple personas that represented different types of clinicians and medical providers in primary care clinics who see diverse chronic pain patient populations in various settings. We used qualitative interviews in this process because it allowed us to ask in-depth questions and worked with providers to elicit different perspectives on these personas. One useful concept that guided our study was the Job-to-be-Done theoretical framework, which suggested that users or customers buy a product to get a job done [28]. Based on this premise, we interviewed primary care providers and asked questions such as what strategies they were using in chronic pain management and why, and the challenges they were trying to solve. These questions provided clues to understand what providers were trying to achieve and to uncover the potential underlying motivations of providers that would potentially influence their engagement with digital therapeutics in routine chronic pain care.

### Study Recruitment

We recruited physicians, nurses, physician assistants, and behavioral health providers, who work in primary care clinics and have treated patients with chronic noncancer pain. We used convenience and snowball sampling for the recruitment of participants within the WWAMI (Washington, Wyoming, Alaska, Montana, Idaho) region Practice and Research Network (WPRN), a regional primary care practice-based research network, and among primary care providers with whom study team members collaborated with in the past. Study outreach focused on recruiting a broad range of provider specialties and roles within primary care clinics and a breadth of practice types and locations. Providers including 10 clinic champions from 11 clinics located in Washington, Idaho, Alaska, Montana, Kansas, and Colorado were invited to participate via email and asked to recruit other providers within their clinics and professional networks. These clinics represented a mix of urban or rural serving, residency training, and nontraining sites. We were not able to collect data on the total number of invitations that were subsequently sent by initial invitees, but everyone who agreed to participate completed their interviews. This study followed the Consolidated Criteria for Reporting Qualitative Research guideline [29].

### Ethics Approval

The study was approved by the University of Washington Human Subjects Division (#STUDY00012442). Participants provided informed consent prior to their interviews. To protect participants’ confidentiality, no direct identifiers were used in the interviews and the participants were assigned with subject codes. Participants received a US $100 gift card for their participation in the study.

### Data Collection

Semistructured interviews with providers in primary care were conducted remotely between July and October 2021. The interview guide (see Multimedia Appendix 1) was developed based on the Job-to-be-Done theoretical framework [28] and chronic pain literature by our research team that consisted of a family medicine physician (YZ), 2 primary care psychologists with expertise in behavioral interventions for chronic pain (KM and KAS), 3 public health researchers (RG, MP, and BLM), and 3 experts or developers in digital therapeutics (SMZ, DW, and JM). Participants were asked to share their goals and priorities in chronic pain management, experiences with challenges and strategies they used, and their perceptions of applying digital therapeutics in clinical practice. RG (a nonclinician researcher) conducted the 1-hour interviews in English either over a videoconferencing platform or over the telephone. All interviews were audio recorded and professionally transcribed for data analysis. Data on participant characteristics were collected via an electronic survey sent to each participant prior to the interview. Interviews were continued until data saturation was reached [30].

### Data Analysis

Transcripts were coded using Dedoose (SocioCultural Research Consultants LLC, Version 9.0.17.) [31] and analyzed using a thematic analysis approach [32]. Two authors, RG and MP, independently coded the transcripts. Our initial codebook had first-level codes like “strategies” and “challenges” from our research question and second-level codes, “managing expectations,” “utilizing specialists,” “obtaining patient buy-in,” and “lack of social resources,” to capture key phrases or responses to our interview questions. After coding the transcripts, RG, MP, and KM used the Job-to-be-Done theoretical framework to help interpret and group the codes that were shown to achieve similar functions of the provider’s job in managing chronic pain. For instance, second-level codes “managing expectations,” “obtaining patient buy-in,” and “goal-setting” could be captured by a higher level code “interactions with patients.” Once all codes were categorized, RG, MP, and KM examined the themes occurring within and across the coding hierarchy. Cross-cutting themes that represented distinct provider motivations (ie, what they were trying to achieve) in chronic pain management were identified. All members of the research team discussed the themes and reached a consensus. To enhance the rigor and trustworthiness of the data, multiple coders were used, and they met frequently to review memos and coding. An audit trail was also kept to document decisions.

## Results

### Participant Characteristics

We completed individual interviews with 11 providers from 4 primary care clinics in Washington and Colorado (see Table 1). Most providers were from clinics affiliated with a large academic health system or in federally qualified or community health centers in urban areas. The sample included 7 primary care physicians, 2 behavioral health providers, 1 physician assistant, and 1 nurse practitioner. They spent an average of 12.44 (SD 8.6) years in primary care. All providers had seen patients with chronic pain in their practice.

**Table 1:** Participant characteristics (N=11).^a^

Characteristics		Participants, n (%)
**Roles**		
	Primary care provider	7 (64)
	Behavioral health provider or therapist	2 (18)
	Physician assistant	1 (9)
	Nurse practitioner	1 (9)
**Degrees**		
	MD	6 (55)
	PhD	1 (9)
	MPH	1 (9)
	Others (MSW, LICSW, JD, PA-C, APRN, and LMFT)	5 (45)
**Specialty**		
	Family medicine	8 (73)
	Behavioral health	2 (18)
**Clinic type**		
	Academic health center	8 (73)
	Hospital-affiliated	2 (18)
	Federally qualified or community health center	1 (9)
**State**		
	Washington	10 (91)
	Colorado	1 (9)
**Clinical practice per week (hours)**		
	10-20	4 (36)
	20-30	2 (18)
	30-40	3 (27)
	>40	2 (18)
**Sex**		
	Female	10 (91)
	Male	1 (9)
**Age range (years)**		
	25-34	2 (18)
	35-44	5 (45)
	45-53	1 (9)
	55-64	2 (18)
**Race**		
	White	8 (73)
	American Indian or Indigenous American	1 (9)
**Ethnicity**		
	Hispanic	0 (0)
	Non-Hispanic	10 (91)

^a^One missing case for degree, specialty, clinic type, age, and race; 2 missing cases for years in primary care; 1 declined to answer for race and ethnicity.

### Themes

Participants shared numerous experiences of strategies and challenges when caring for patients with chronic pain. Using the Job-to-be-Done theoretical framework [28], we summarized the data into 4 themes that reflected what primary care providers were trying to achieve with patients in chronic pain management. We described each of the 4 themes below.

#### Strengthen Patient-Provider Alliance

Providers believed that the patient-provider alliance in chronic pain management requires empathizing with patients about their lived experiences, eliciting patients’ goals, and providing education on chronic pain and treatment options. A participant said:

The first part is just trying to understand what they enjoy doing. [...] to see if I can find out what they like. Then, when did they last do that, what have been some of the barriers that they can think of, and then just talking about the cycle of psychoeducation about what happens with depression [...] Just try to validate that, even though it's seemingly simple, it's one of the hardest things in the world to get started doing something.

Behavioral health provider

Some providers took notes about patients’ personal details (eg, name of a patient’s family member) as reminders for future visit discussions. Other providers asked patients their favorite pastime that they have avoided due to pain and found ways to help patients re-engage in activities that are meaningful to them. Providers emphasized the importance of educating patients on the biological, psychological, and social components of pain and the variety of treatment options that are evidence-based, safe, and appropriate to patients. These conversations discussed the benefits and risks of pharmacological treatments and normalized nonpharmacological treatments.

What providers found challenging was the brevity of 15- to 20-minute appointments that typically did not allow them to do all of the things mentioned above and to gain trust and mutual understanding with patients. Providers felt pressured to see a high volume of patients, which made conversations with patients about their lives, goals, and treatment plans difficult to achieve.

Providers reported lacking the time, skills, and resources needed to process patients’ distress and repair their alliance when there is misalignment between patients and their expectations. A participant described it this way:

[The health system] wants me to be productive as a PCP and see so many patients per day, which limits the amount of time that I can spend per visit with each patient. So having adequate time to provide this education, to sit and to listen to the tears and the fear, and then try to manage that with the patient in a patient centered, compassionate way is hard. So, time is a big issue.

Primary care provider

Providers relayed that they occasionally feel conflicted about maintaining boundaries with opiate prescribing (eg, dosing or tapering) when time constraints do not allow for comprehensive education and realistic goal-setting conversations with patients for a shared decision-making process.

#### Provide Team-Based Care

Considering the complexities of chronic pain diagnosis and management, primary care providers relied on support from pain specialists, physical therapists, behavioral health providers, and pharmacists. Providers described seeking consultations from multidisciplinary team members for diagnostic clarification and ideas on managing patient’s pain. In addition, they referred patients to work with certain specialists for specific treatment goals in order to provide whole person care. Primary care providers noted that their ability to use multidisciplinary team support was limited by the availability of in-clinic consulting specialists and structured time for consultation. Only a few providers reported access to social work, behavioral health, and pharmacy services in their clinics. One participant mentioned that their clinic used to have allotted time for in-person consultation with a pain specialist on a monthly basis:

We had a pain consult guy come to [clinic] for a while. He would come about once a month, and it was a set thing on the schedule. The front desk would often try to schedule your chronic pain patients for that spot. He really thought of all the angles, he was a nice resource to have, but that's not a thing anymore, I don't know what happened with that.

Nurse practitioner

Providers desired regular interdisciplinary team meetings to review complex cases and gain new perspectives and suggestions on how to support their patients.

#### Track and Monitor Patients’ Progress

Multiple providers raised concerns about the lack of standardized clinic processes and structures in identifying, tracking, and monitoring patients’ progress along a care trajectory. Providers shared that they relied on patients to self-monitor their pain symptoms, changes in activity, and factors impacting those changes over time. A participant said:

Ideally, I use scales. I have done that in the past, but in practicality that doesn’t always happen, doesn’t always fit in. So a lot of it’s just kind of freeform conversation

Primary care provider

Some providers mentioned they had experiences recommending mobile apps to patients for tracking and self-monitoring health conditions such as blood pressure, glucose levels, and menstrual cycles and used the data for follow-up visits; however, none of them knew about apps specific for chronic pain. Few providers also expressed their concerns about how to educate patients to use apps for self-monitoring. Rarely did providers use standardized assessments for pain in visits because they think pain conversations needed to be tailored to each patient. However, at the population level, providers were aware of the potential pitfalls of not tracking patients’ symptoms in a standardized way.

In addition, providers said there were no support structures or streamlined processes in the clinics to coordinate follow-up care for patients with chronic pain. Many providers developed “workaround” solutions to track patients’ appointments and remind themselves of scheduling follow-up visits with patients. For instance, some providers described scheduling patients themselves for follow-up at the end of a visit or providing a direct hand-off to the schedulers with instructions on the timeframe for the patient’s next visit. Other providers manually reviewed their patient panels from time to time and reached out to patients who missed appointments or required follow-up. The nurse practitioner said:

I have an inbox, and I keep things there if there's results or a person that I want to follow up on, or I don’t want to forget about. I will go through that every once in a while.

Although the process of monitoring and tracking patients and visits was individualized to provider preferences, providers considered this to be time-consuming and burdensome for themselves and the patients, and left much room for compromising patients’ care. Providers added that the electronic health record (EHR) systems were not user-friendly to providers for documentation and tracking, which further complicated their panel management.

#### Address Social Determinants of Health

Providers considered the overall impact of social determinants of health on the chronic pain experience for patients. As part of treatment planning, almost all providers inquired about potential factors and barriers that impact patients’ access to care. Once barriers were determined, providers reached out to other clinic members and the patient’s support system (eg, family) to find ways to address the barriers. For instance, a provider shared they connected patients with in-clinic social workers for referrals to community resources meeting basic needs. Another provider sought interpreter services for multilingual patients to overcome language barriers during treatment. Despite the initiatives, providers recognized some social and systemic barriers remain persistent and difficult to address. Providers specified the lack of health insurance coverage and access to reliable transportation as common barriers for patients in accessing nonpharmacological treatments. A participant said:

Then also sustainability, like how realistic is this practice for this patient. Especially at [clinic] a lot of our patients don't have access to adjunct chronic pain things like acupuncture, or even getting to physical therapy, or swimming, or things like that. So really kind of considering the socioeconomic status and resources [of] that patient.

Physician assistant

They also mentioned factors like being unhoused and inadequate access to nutritious foods exacerbate chronic pain and delay treatment. When the idea of using digital technologies to augment care was discussed, some providers raised concerns about the inequitable access of these technologies for underserved patients, including those who speak a primary language other than English, have low digital literacy, and have unstable internet access.

## Discussion

### Principal Findings

Drawing upon providers’ reported experiences of strategies and challenges, this qualitative, human-centered design study revealed provider motivations in chronic pain management that would have implications to the development of digital therapeutics tailored to primary care settings. We found that primary care providers were motivated to (1) strengthen patient-provider alliance, (2) provide team-based care, (3) track and monitor patient’s progress, and (4) address social determinants of health in chronic pain management. Specifically, providers desired additional resources to improve patient-centered communication, pain education and counseling, and goal setting with patients. Providers also requested greater accessibility to multidisciplinary care team consultations and nonpharmacological pain treatments. When managing chronic pain at the population level, providers need infrastructure and systems to systematically track and monitor patients’ pain and provide wraparound health and social services for underserved patients. Given these findings, this study underscored the importance of designing digital therapeutics that would engage and support primary care providers in overcoming challenges to fulfill their various motivations when caring for patients with chronic pain. Below, we offer 4 digital therapeutic feature areas supported by the findings and literature that might address provider challenges in meeting those motivations.

### Recommendations

First, patient-provider alliances can be strengthened through the inclusion of patient-generated data and the development of communication interfaces in digital therapeutics [33]. The inclusion of patient-generated data may improve the disconnect between providers and patients described by participants, as this disconnect often occurred when providers did not have time to obtain in-depth information about patients’ lived experiences and the emotional impact of their pain [34,35]. Digital therapeutics can capture patient-generated data over time on patients’ physical, social, behavioral experiences, and their environment. These data could help providers make accurate diagnoses, engage effectively with patients for treatment recommendations, and connect patients to community resources for basic needs [34,36]. Interactive digital communication interfaces may help primary care providers and patients streamline their chronic pain conversations in brief clinic visits and facilitate goal setting. Providers can leverage digital tools with patients to prioritize clinical concerns, set the agenda for visits, personalize their chronic pain discussions based on patient needs, and provide feedback on treatment progress—practices that have been found to help efficiently address a multitude of clinical issues in visits and improve patient engagement [37].

Second, digital therapeutics that integrate seamlessly with care delivery systems are crucial to population health management for chronic pain [33,38]. Our findings and prior studies have shown that current approaches to tracking and monitoring of pain and other co-occurring conditions in primary care practices were often only applied to patients on long-term opioid therapy, motivated by the practice goal of opioid-prescribing reduction rather than comprehensive, multidisciplinary pain care [39]. The inclusion of patient-generated data from digital therapeutics, which can be imported confidentially to the EHR, could be beneficial in three ways: (1) provide valuable assessment data for providers to tailor care for each individual patient; (2) expand the remote patient monitoring capabilities to the whole patient population experiencing chronic pain; and (3) connect patients to support, education, and resources to help manage their pain in between visits [40].

Third, the emerging evidence on digital therapeutics that are developed using evidence-based behavioral interventions for chronic pain could be a promising alternative to providing patients with more support and treatment options, especially when a shortage of pain specialists and health care workers who deliver nonpharmacological pain treatments limit access to care [11,14,18,19,22]. Digital therapeutics, available outside of the confines of a clinical visit, may complement patient education delivered by primary care providers, standardize delivery of care, and increase access to treatment information and choices that facilitate shared understanding and decision-making between providers and patients, which is crucial to providing guideline-concordant chronic pain care [13]. Digital therapeutics also allow for provider-facing features where primary care providers can access remote experts in chronic pain management and coordinate team-based care for patients [38].

Fourth, our findings suggested that chronic pain is complicated by social determinants of health, and reducing disparities in access to care for under-resourced populations is a top concern for primary care providers. One study has demonstrated the feasibility of mobile apps and fitness trackers to engage disadvantaged and multilingual patients to collect patient-generated data that can be integrated into the EHR to improve chronic disease management in primary care [36]. Although the overall smartphone ownership in America has risen to 85% in 2021 as compared to 35% in 2011, which includes a growth in smartphone ownership among Americans with lower income [41], the digital divide still persists in technology adoption between families living with US $100,000 or more a year versus those living US $30,000 or less per year [42]. The question of how digital therapeutics can be equitably used for chronic pain management based on patients’ accessibility to the internet, smartphone ownership, preferred languages, levels of literacy, and affordability warrants further investigation, especially when persistent economic, educational, and racial or ethnic disparities in chronic pain are well documented in the literature [13,43]. Chronic pain disproportionately affects people with multiple chronic conditions, who in turn are affected by higher levels of poverty and lower access to care. Digital therapeutics for chronic pain will have more equitable reach if developed with an eye toward using platforms and solutions that require lower sophistication of device and internet access.

In addition to designing features that could meet providers’ motivations, special attention should also be given to the implementation of digital therapeutics in health care systems. Prior studies have suggested possible logistical and workflow challenges, such as training providers to use digital apps with patients in clinical visits, creating a workflow to assist patients in enrolling and setting up digital app programs, and designating personnel and time to review patient-generated data from the digital therapeutics [44,45]. These challenges, if not addressed adequately, could heighten the existing burden of providers and lead to low provider engagement. Given each primary care practice is unique and has its own characteristics, there is no one-size-fit-all solution to the challenges of implementing digital therapeutics. This study findings could be useful in identifying value propositions that help gain buy-in from providers and clinics, as well as potential implementation factors that inform the design of digital therapeutics to be feasible and acceptable to primary care.

### Limitations

Despite our outreach attempts, multiple challenges arose for health care providers to participate in research during the COVID-19 pandemic. Many providers who were unable to participate in the study responded that the reasons for their inability to participate included a lack of time due to clinical duties from COVID-19, provider shortages in their clinics, and other competing priorities. The small sample size of 11 participants who were recruited from 4 clinics in urban and academic health systems in the states of Washington and Colorado may not be generalizable to all primary care providers, and there might be bias in participants who volunteered to join the study. Also, many of our participants were female, non-Hispanic, and middle-aged—provider characteristics that have shown influences on some pain management decisions and opioid-prescribing patterns [5,46]. Nevertheless, the participants represented providers with different professional roles, varying years of experience in primary care, and working with diverse patient populations including those with minoritized racial or ethnic and low-income backgrounds. In this regard, the study findings still captured important interdisciplinary and various frontline perspectives that are needed for developing chronic pain-related digital solutions in primary care.

### Conclusions

Based on our findings, to engage primary care providers, digital therapeutics for chronic pain management need to support and strengthen the patient-provider alliance, team-based care, systematic tracking and monitoring, and addressing social determinants of health. Leveraging digital therapeutics in a feasible, appropriate, and acceptable way to aid primary care providers in chronic pain management may require multimodal features that address provider motivations at an individual care and clinic or system level.

## Appendix

Multimedia Appendix 1 Interview guide.

## Abbreviations

EHR: electronic health record
WPRN: WWAMI region Practice and Research Network
WWAMI: Washington, Wyoming, Alaska, Montana, Idaho

## Data Availability

The data presented in this paper will be shared on reasonable request to the corresponding author.
