# Supplementary material for: Developing Digital Therapeutics for Chronic Pain in Primary Care: A Qualitative Human-Centered Design Study of Providers’ Motivations and Challenges
Source: JMIR Form Res. 2023 Feb 3;7:e41788. doi: 10.2196/41788 (PMC9938436; doi:10.2196/41788)
Supplement: Multimedia Appendix 1 [file formative_v7i1e41788_app1.docx]

**Appendix 1.** Interview Guide

1. What are your goals or priorities when caring for patients with chronic pain (CP)?
2. How do you approach your care and treatment for patients with CP?

- What is working well in your care for these patients?
- What has helped you provide care?
- What do you do when your goals are not aligned with patients’ goals?
- How do you create buy-in for other treatment models?

1. What is challenging in your care for these patients? Why?

- What would help you treat patients better?

1. What do you assess, track, and monitor for patients with CP?

- What is working well in your monitoring and following up with patients with CP?
- What is challenging in your monitoring and following up with patients with CP?

1. What have been your experiences with panel management?

- What types of panel(s) do you have in your practice?
- How are these panels chosen or decided?
- What would help you/clinic to consider doing panel management for patients with CP?
- What are some reasons for not doing panel management in your practice?

1. What have been your experiences with recommending smartphone app-based digital therapeutics to patients in clinical practice?

- What apps have you recommended to patients? And why?
- How do patients receive your recommendation?
- How do you use the app(s) with patients in your practice?
- What do you like about recommending mobile apps to patients?
- What is challenging about recommending mobile apps to patients?
